# Cross-cultural adaptation, reliability and validity of the Turkish version of the Chronic Venous Disease Quality of Life Questionnaire (CIVIQ-20)

**DOI:** 10.1186/s40064-016-2039-2

**Published:** 2016-03-31

**Authors:** Özlem Cinar Özdemir, Eda Tonga, Agah Tekindal, Yesim Bakar

**Affiliations:** The Institute of Health Sciences, School of Physical Therapy and Rehabilitation, Abant İzzet Baysal University, 14130 Bolu, Turkey; The Faculty of Health Sciences, Marmara University, Istanbul, Turkey; Department of Biostatistics, Izmir University, Izmir, Turkey

## Abstract

**Background:**

Chronic venous disease (CVD) is a well-defined and known disorder which impact on related-health quality of life (QoL). The Chronic Venous Insufficiency Quality of Life Questionnaire (CIVIQ) is a disease-specific instrument to measure the impact of chronic venous insufficiency on patients’ lives. The purpose of this study is to cross-culturally adapted the Chronic Venous Disease Quality of Life Questionnaire (CIVIQ-20) for Turkish-speaking patients and determine the psychometric properties of reliability, validity and factor structure in a Turkish population with CVD.

**Methods:**

The CIVIQ-20 was translated into Turkish and culturally adapted using a double forward–backward protocol according to established guidelines. Individuals (n = 140) with venous diseases completed the CIVIQ-20, Venous Insufficiency Epidemiological and Economic Study (VEINES-QoL/Sym) and Nottingham Health Profile (NHP) questionnaires at baseline and 1 month later.

**Results:**

Cronbach’s α value was 0.93. Test–retest reliability was determined as moderate (ICC_2:1_ = 0.80). There was a significant correlation between CIVIQ-Tk and Nottingham and VEINES-QoL total scores (Nottingham 1: r = 0.770; p < 0.00, Nottingham 2: r = 0.7000; p < 0) (VEINES-QoL: r = −0.574; p < 0.00, VEINES-QoL 2: −0.592, p: 0.00). The measurement error were calculated from SEM and MDC_90_. The SEM was 2.63 and the MDC_90_ was 5.79. Exploratory factor analysis demonstrated a three factor structure that explained 56.32 % of total variance.

**Conclusion:**

The CVIQ-20 Turkish is a reliable and valid instrument for Turkish speaking patients with chronic venous insufficiency.

## Background

Chronic venous insufficiency (CVI) is defined as a very common medical condition that is related to the venous system at the lower extremities involving various pathologies and subjective symptoms such as pains, cramps, irritability in legs, edema, itchiness and skin changes (Gulati 2008; Bergan et al. 2006). It is a common disease and in the Western countries approximately 62.1 % of females and 49.1 % females are affected by venous symptoms (Pannier-Fischer and Rabe 2003). About half of the general adult population shows the signs of CVI while about a quarter of them have lower-extremity varicose veins (Evans et al. 1999). In Turkey, however, the CVI condition is reported to affect 20–25 % of women and 10–15 % of men (Akbulut et al. 2009).

Due to the appearance of varicose veins, CVI symptoms and complications, the CVI patients may have significant levels of health-related quality of life impairment (HRQoL) (Launois et al. 1996). The studies show that there is a rapid deterioration in quality of life (QoL) in persons with venous disease (Kurz et al. 2001; Chiesa et al. 2007). Measurement of QoL that enables a better understanding of the effects of the disease is significant in terms of evaluating the disease (Garratt et al. 1993). Although the venous diseases have high prevalence rates, they have not been sufficiently studied up until now due to the limited number of studies conducted on measuring the quality of life (Smith et al. 2000; Kaplan et al. 2003). Disease-specific and general quality of life scales have been developed to evaluate these cases. Today, numerous proven result measures are being used and they are divided into generic and disease-specific QoL tools. While general quality-of-life scales can be used in general evaluation they fail to meet the CVI-specific conditions. In the treatment evaluation, however, care effects and disease related effect determination as well as the quality-of-life scales have been proven to be more effective. It is recommended that the generic and disease-specific QoL instruments are combined in order to integrate the deficiencies of the generic tools (Lamping et al. 2003; Kahn et al. 2004).

36-Item Short Form Health Survey (SF-36) is a commonly-used and recognized generic quality-of-life tool. The Nottingham Health Profile (NHP) is another generic tool which was developed to be applied in various conditions. Aberdeen Varicose Vein Questionnaire (AVVQ), the Chronic Venous Insufficiency Quality of Life Questionnaire (CIVIQ) and the Venous Insufficiency Epidemiological and Economic Study (VEINES-QoL/Sym) questionnaire are the most frequently used disease-specific tools for varicose veins and CVI (Vasquez and Munschauer 2008). In Turkey, only the VEINES quality-of-life questionnaire is used to measure the QoL in patients who show the symptoms of CVI.

Launois et al. developed the CIVIQ in 1996 (Launois et al. 1996). It has been established that this disease-specific questionnaire is valid and highly reproducible with great internal coordination and high responsiveness rate. It has also been established that it is a precious tool in both clinical practice and trials for evaluating improvement in patients’ HRQoL (Lozano and Launois 2002). The source questionnaire for CIVIQ-20 was confirmed in French. Although there are Spanish, Dutch and Greek versions of the test available, it has not been translated into other languages (Launois et al. 2013).

In order to measure the QoL in patients with CVI symptoms in Turkey, only the VEINES quality-of-life questionnaire is used. The present study is planned to establish the reliability, validity and responsiveness of the Turkish version of the CIVIQ in CVI patients. Thus, the present study aims to adapt the VEINES-QoL cross-culturally for Turkish-speaking patients and to define the clinical characteristics of reliability, criterion validity, internal consistency, measurement mistakes and factor structure in patients with Chronic Venous Disease.

## Methods

### Study subjects

All the consecutive patients who consulted the departments of vascular surgery of the Abant Izzet Baysal University for varicose veins were asked to participate in this study. Patients aged 18 years old who had CVI in CEAP C stages C3–C6 and who had the ability to fill in two quality of life questionnaires were included in the study. Patients who had no reflux during venous ultrasound examination were excluded from the study. The study was approved by the Non-Interventional Clinical Researches Ethical Committee of Abant Izzet Baysal University. Informed consent forms were obtained from all individual participants included in the study.

### Procedure

165 patients with CVI were found eligible and invited to participate in this study. Before their outpatient appointment, all patients were asked to fill out both of the disease-specific QoL instruments, the VEINES-QoL and CIVIQ-20 and in addition one general QoL tool, the NHP. 140 patients received generic and disease specific HRQoL (CIVIQ-1), and were asked to fill it out in complete self-administration. After a period of 1 month, the patients were asked to fill out the tests for test–retest reliability, 120 of whom returned the CIVIQ-2. The same physiotherapist collected all tests.

### Questionnaire

CIVIQ-20 is a QoL survey that contains 20 questions and is specific for CVI, encompassing four QoL domains: physical (items 5, 6, 7 and 9), psychological (items 12, 20) and social impairment (items 8, 10 and 11) and the severity of pain (items 1, 4). There is a 5-point answer rating in each question, according to which higher scores indicate more severe impairment. There is a score varying from 0 to 100 in the CIVIQ-20 where the higher scores indicate better QoL (Launois et al. 1996).

VEINES-QoL/Sym measures the impact of CVI on symptoms and quality of life (QoL) from the patient’s perspective. The validity and reliability of the Turkish version of the VEINES-QoL/Sym was performed by Kutlu et al. (2011). The 26 items and 2 dimensions of the VEINES-QoL/Sym measure the impact of CVI on symptoms and QoL from the patient’s perspective. The first 10 cover the symptoms (fullness of lower extremities, pain, swelling, night cramps, heat/burning sensation, restless legs, itching, tingling/stinging sensation, and throbbing) in 5 different frequencies (always, a few times a week, once a week, once in a few weeks, and never). Limitations in daily activities (9 items), time of day of greatest intensity (1 item), and change over the past year (1 item), and psychological impact (5 items) are covered by the QoL scale with 2- to 7-point response scales of intensity, frequency, or agreement. Venous symptoms were assessed based on the symptoms that are part of the VEINES-QoL/Sym. Each item score varies between 0 and 6, according to which high scores indicate better outcomes in VEINES-QoL and VEINES-Sym scales (Kutlu et al. 2011).

Nottingham Health Profile (NHP) is a generic health instrument that is used for the measuring the quality of life. It contains 6 subscales and 38 questions with each assigned a weighted value; the sum of all weighted values in a given item adds up to 100. The 6 subscales include the energy level (EL) subscale containing 3 questions, pain (P) subscale contains 8 question, emotional reaction (ER) subscale containing 9 questions, sleep (S) subscale, containing 5 question, social isolation (SI) subscale containing 5 questions, and physical abilities (PA) subscale containing 8 questions. To obtain a final score in each dimension, and to overcome the issue of having a different number of items in some of the dimensions, each sum was multiplied by 100 and divided by the number of items in the dimension. Possible scores ranged from 0 (all answers of “no” in the dimension, denoting absence of distress) to 100 (all answers of “yes”, denoting maximal distress). High scores indicate worse outcomes. The reliability and validity of the Turkish version of the NHP has been demonstrated in a previous study (Kücükdeveci et al. 2000).

### Translation and cross-cultural adaptation

By means of the suggested double forward backward translation, the CIVIQ questionnaire was translated into Turkish (Launois et al. 2013). The questions and the answer choices of the English version were translated separately by two Turkish native-language translators and thereby the CIVIQ was adapted into the Turkish context. The translators were from a registered translation office and a physiotherapist for better idiomatic and conceptual (rather than literal) equivalence and reliability. Thus, the words and language structure were elementary. The two translators compared and discussed the obtained separate forward translations. The purpose of the stage was to determine and solve the insufficient terms and expressions of the scale which was translated into the target language separately by bringing the experts in the jury together. At the end of this stage, the questionnaire that was translated into Turkish was put into the final form. The two English native-language speakers back translated blindly and separately. For contradictions, the final forms and the original version were compared and a pilot agreement form was completed. In order to obtain a qualitative testing of legibility and understanding, the Turkish CIVIQ was given to 20 patients who had venous disease of the lower limbs. These persons were asked whether they had difficulty in understanding the questionnaire and whether there were complicated expressions. Because of this qualitative questionnaire proved no difficulty with the Turkish CIVIQ, all cultural adaptation procedures were completed and the scale was put into the final form after the corrections were made. It was subsequently administered to the study population to collect data for psychometric analysis.

## Statistics

Descriptive analyses were applied to calculate means and standard deviations of the demographic variables. Distribution and normality were determined by the one-sample Kolmogorov–Smirnov tests (significance >0.05). Construct validity and factor structure were determined from maximum likelihood extraction (MLE) with the a priori extraction requirements being satisfaction of three criteria: scree plot inflection, Eigenvalue >1.0 and variance >10 % (de Vet et al. 2011; Costello and Osborne 2005). The recommended minimum ratio of ten participants-per-item was satisfied (Cronbach 1951). Factor structure was assessed using exploratory factor analysis (EFA) with MLE and Varimax rotation. The three a priori criteria for inclusion of the extracted factors were: Eigenvalues >1, accounting for >10 % of variance and visually determined on the scree plot (de Vet et al. 2005). The internal consistency was determined from Cronbach’s α coefficient (Stratford 2004). Criterion validity was determined through the concurrent use of all QoL instrument. The Pearson’s r correlation coefficient used the criteria of poor (r < 0.49), fair (r = 0.50–0.74) and strong (r > 0.75) (Fabrigar et al. 1999).

Reliability was performed using the Intraclass Correlation Coefficient Type 2:1 (ICC_2:1_) test–retest methodology in the full sample recorded at baseline and 2–4 weeks following a period of no treatment. The sensitivity or error score was determined from the MDC_90_ analysis that was performed as described by Stratford (Stratford 2004). The standard error of the measurement (SEM) was calculated using the formula: SEM = s√(1 − r), where s = standard deviation (SD) of time 1 and time 2, r = the reliability coefficient for the test and Pearson’s correlation coefficient between test and retest values. Thereafter the MDC_90_ was calculated using the formula: MDC_90_ = SEM × √2 × 1.65.

All statistical analyses were conducted using the Statistical Package for Social Science version 17.0 (SPSS 17.0) for Macintosh.

## Results

### Participants

140 patients with CVI was participated in the study. The average age of participants 52.3 ± 13.40 years. The 101 patients were women; 39 were men. The demographic characteristics of subjects were shown in Tables 1 and 2. The variables were normally distributed (p > 0.05). Double forward backward translation of the CIVIQ was conducted without difficulty. Cognitive debriefing interviews were conducted with 5 patients for considering if the questionnaire is understandable and culturally relevant. As a result of cognitive debriefing interviews all patients who participated to the interviews reflected that the questionnaire is easily understandable, readable and culturally relevant.

**Table 1:** Demographic and clinical features of the patients

	X ± SD
Age (years)	52.3 ± 13.4
Height (m)	1.62 ± 12.6
Weight (kg)	81.1 ± 11.6
BMI (kg/m^2^)	30.2 ± 4.3

**Table 2:** Socio-demographic characteristics of the study population

	n	%
Gender		
Woman	101	72.1
Man	39	27.9
Educational status		
Illiterate	2	1.4
Primary level	72	51.4
High school level	45	32.1
University	21	15
Occupation		
Housewife	65	46.4
Worker	35	25
Retired	40	28.6
CEAP		
C3	119	85
C4	17	12.1
C5–C6	4	2.9

### Internal consistency, test–retest reliability, criterion validity and error score

Cronbach’s α value was 0.93 which indicate a high internal consistency of CIVIQ Turkish. Test–retest reliability was determined as moderate (ICC_2:1_ = 0.80). There was a significant correlation between CIVIQ Turkish, Nottingham and VEINES-QoL total scores (Nottingham 1: r = 0.770; p < 0.00, Nottingham 2: r = 0.7000; p < 0) (VEINES-QoL r = −0.574; p < 0.00, VEINES-QoL 2: −0.592, p: 0.00). The value of this correlation means a strong criterion validity. The measurement error were calculated from SEM and MDC_90_. The SEM was 2.63 and the MDC_90_ was 5.79.

### Factor analysis

**Fig. 1:**
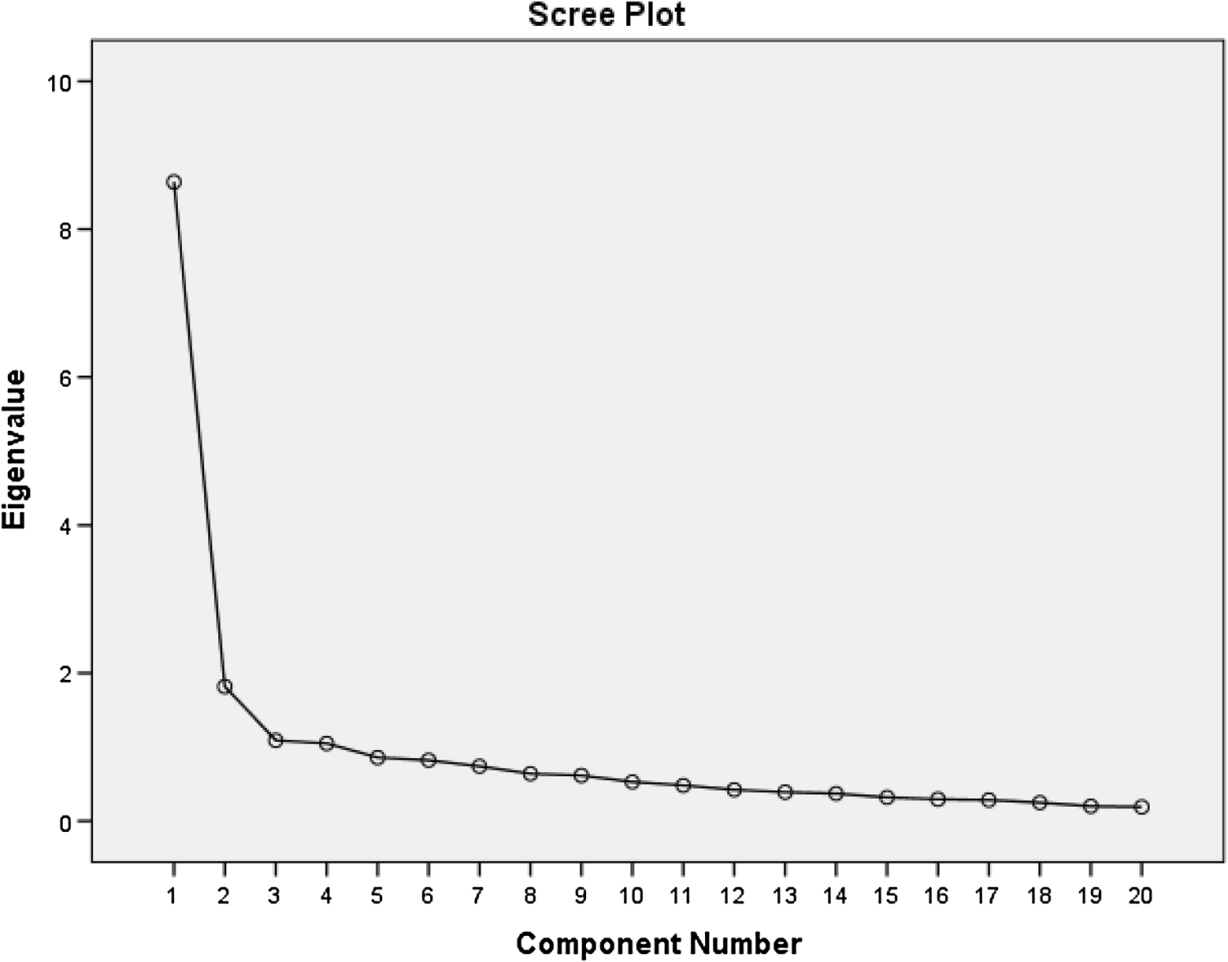
Scree plot of the factor analysis

The correlation matrix for the CIVIQ Turkish was determined as suitable from the Kaiser–Meyer–Olkin values (0.66) and the Bartlett’s Test of Sphericity (p < 0.001). This indicated that the correlation matrix was unlikely to be an identity matrix and was therefore suitable for MLE. The scree plot (see Fig. 1) indicated a three-factor solution. The factor analysis revealed a satisfactory percentage of total variance explained by the three factor at 56.32 %.

The factor analysis revealed four factors with Eigenvalues >1. Four factors accounted for >10 % variance and the scree plot occurred at the third point (Fig. 1). The item loading for the three-factor solution for the MLE method and average score for each item are shown in Table 3.

**Table 3:** Factor analysis loadings of the CIVIQ-20

Items	Factors		
	1	2	3
1. Pain in legs	0.500	0.113	0.535
2. Interferes with work	0.691	0.172	0.247
3. Sleeping poorly	0.257	0.120	0.800
4. To stand for a long time	0.730	0.212	0.156
5. To climb stairs	0.785	−0.008	0.069
6. To crunch/to kneel	0.726	0.243	0.181
7. To walk briskly	0.743	0.067	0.130
8. To travel by car/bus/plane	0.602	0.195	0.237
9. To do the housework	0.813	0.081	0.254
10. Going to parties	0.697	0.138	0.410
11. To perform sports activities	0.751	0.183	0.200
12. Feel on edge	0.275	0.517	0.462
13. Get tired easily	0.601	0.480	0.157
14. Feel like a burden people	0.225	0.437	0.385
15. I feel weaker and stiffer	0.480	0.544	−0.090
16. Embarrassed to show legs	0.008	0.768	0.179
17. Easily irritable	0.031	0.681	0.255
18. Impression of being disabled	0.379	0.681	0.120
19. Difficulty getting up in the morning	0.211	0.210	0.680
20. Do not feel like going out	0.193	0.193	0.140

## Discussion

The CIVIQ is used in clinics in Turkey with simply translated versions (without formal methodology). However, there is no study on culturally adaptation and linguistic validation of CIVIQ in Turkish speaking population. Therefore, we decided to do a study that aims to adapt the CIVIQ cross-culturally for Turkish-speaking patients. The procedure of translation and cross-cultural adaptation were easily conducted with the consideration of international guidelines. The procedure is also similar with methodology of other studies that adapt the patient reported questionnaires in Turkey (Tuygun et al. 2012). The face validity was observed through the cognitive debriefing interviews and the translation process The Turkish version of CIVIQ questionnaire is self-administered, simply completed and short lasting according to cognitive debriefing interviews. There was no need to extract any item, add a new item or modify any item. Launois et al. reviewed the all linguistic validation studies of CIVIQ in different countries. According to Launois study there were some linguistic or culturally adaptations in items in some countries (Launois et al. 2013).

The Cronbach’s α coefficient at 0.93 showed strong internal consistency. This result similar with Dutch and Greek versions (>0.90). That seems the questionnaire is strongly reliable because the Cronbach’s α value >0.70 but a little excessive similar with Dutch and Greek versions. The test–retest reliability (r = 0.80) was high and comparable with the findings of Dutch version (ICC_2:1_: 0.86) (Biemans et al. 2011; Erevnidou et al. 2004).

The factor structure was shown as a 3-factor structure (physical, pain, psychological) under the a priori criteria similar with Dutch version. Differently four quality of life dimensions (Physical, psychological, social, pain) used in original CIVIQ-20. The extracted first factor has a satisfactory percentage of total variance explained by the one factor at 30.6 %. While the Dutch version’s extracted of the first factor accounted for 43.7 % of the variability of the CIVIQ. There is no item that loads under 0.20 but in Greek version there is one item loaded <0.20.

Strengths of the study included the standardized methods employed for all psychometric procedures and the cross-cultural adaptation process. Importantly, this is the second study to adapt a CVI questionnaire to the Turkish language for general use. The limitations were the lack of longitudinal data regarding other psychometric properties including responsiveness.

## Conclusion

The CIVIQ Turkish demonstrated a 3-factor structure and is a reliable and valid instrument. The CIVIQ Turkish in its present form consists of simple and easily understood language which may enable it to be used to assess chronic venous insufficiency in Turkish speaking patients.

## Abbreviations

CVI: chronic venous insufficiency
HRQoL: health-related quality of life
SF-36: 36-Item Short Form Health Survey
NHP: Nottingham Health Profile
AVVQ: Aberdeen Varicose Vein Questionnaire
CIVIQ: Chronic Venous Insufficiency Quality of Life Questionnaire
VEINES-QoL: Venous Insufficiency Epidemiological and Economic Study
BMI: body mass index
kg: kilogram
m: metre
